# Glycine neurotransmission: Its role in development

**DOI:** 10.3389/fnins.2022.947563

**Published:** 2022-09-16

**Authors:** Rocío Salceda

**Affiliations:** Departamento de Neurodesarrollo y Fisiología, Instituto de Fisiología Celular, Universidad Nacional Autónoma de México, Juriquilla, Mexico

**Keywords:** glycine, glycine receptor, development, neurotransmission, GlyR isoforms

## Abstract

The accurate function of the central nervous system (CNS) depends of the consonance of multiple genetic programs and external signals during the ontogenesis. A variety of molecules including neurotransmitters, have been implied in the regulation of proliferation, survival, and cell-fate of neurons and glial cells. Among these, neurotransmitters may play a central role since functional ligand-gated ionic channel receptors have been described before the establishment of synapses. This review argues on the function of glycine during development, and show evidence indicating it regulates morphogenetic events by means of their transporters and receptors, emphasizing the role of glycinergic activity in the balance of excitatory and inhibitory signals during development. Understanding the mechanisms involved in these processes would help us to know the etiology of cognitive dysfunctions and lead to improve brain repair strategies.

## Introduction

The central nervous system (CNS) development is a long process that starts early during embryogenesis and takes years to be completed in humans. The initial steps involve precise coordination of cell proliferation, differentiation, and cell migration. A tight control of these processes is achieved by integration of the intrinsic genetic program with the extracellular signals present in the environment (Platel et al., 2010; Caronia-Brown and Grove, 2011; Káradóttir and Kuo, 2018; Ali and von Gall, 2022). Many factors have been identified as regulators of neurogenesis; among these, extracellular molecules, neurotransmitters, and their receptors have been found to be present in the developing brain well before synaptogenesis occurs (Casanova and Trippe, 2006; Metzger, 2010; Carulli and Verhaagen, 2021), suggesting that they could mediate signaling unrelated to classical neurotransmission.

Early neurotransmitter signaling has been implicated in a range of developmental processes, such as differentiation, migration, neurite outgrowth, axon pathfinding, synaptogenesis, and survival of nascent neurons (Nguyen et al., 2002; Heng et al., 2007; Platel et al., 2010; Spitzer, 2012). The inhibitory neurotransmitter, glycine and its receptors are not only present but also functional in the developing brain before synaptogenesis occurs, suggesting their involvement in development (Chalphin and Saha, 2010).

## Adult glycinergic neurotransmission

In addition to its role in cell metabolism, being the structurally simplest amino acid, glycine, acting through ionotropic receptors, also serves as an important and widely distributed inhibitory neurotransmitter that is most prominently expressed in adult brainstem, spinal cord, and retina of animals from several phyla (Aprison and Werman, 1965; Werman et al., 1968).

The biosynthesis of glycine for its use in neurotransmission is mediated by the serine hydroxymethyl transferase, which uses pyridoxal phosphate and tetra hydrofolate as cofactors of the reaction. In nervous system, glycine is also synthetized by the glycine synthase (glycine cleavage, GCS) enzyme, which catalyzes a readily reversible reaction between carbon dioxide, ammonium ion, N5- N10-methylene tetrahydrofolate, NADH and a proton to produce glycine, tetrahydrofolate and NAD^+^ (Daly and Aprison, 1974). Immunohistochemistry and *in situ* hybridization studies in rats revealed that the glycine cleavage enzyme is also expressed in embryonic neural stem/progenitor cells, neuroepithelial cells, and astrocytes (Ichinohe et al., 2004).

Glycinergic transmission requires of high-affinity specific transporters GlyT1 and GlyT2 for the reuptake of glycine from the synaptic cleft into cells. These proteins are members of the Na^+^/Cl^–^ dependent neurotransmitter transporter family, with GlyT1 expressed predominantly in glial cells and GlyT2 by neurons (Zafra and Giménez, 2008; Eulenburg and Gomeza, 2010). In addition to glycinergic transmission, GlyT1 can modulate glutamatergic neurotransmission through NMDA receptors, supporting its role in brain function and in various diseases (Marques et al., 2020).

Glycine action is mediated by a strychnine-sensitive ligand gated chloride channel glycine receptor (GlyR), which belongs to the cys-loop ligand –gated ion channel superfamily that are composed of five protein subunits that form homomeric or heteromeric pentamers assemble around a central ion-conducting pore (Langosch et al., 1988; Schmieden et al., 1992; Betz et al., 1993; Lynch, 2004). GlyRs are anchored postsynaptically by gephyrin, which binds to the β receptor subunit and tubulin, resulting in the receptor clustering (Feng et al., 1998; Kneussel and Betz, 2000). Four α subunits (1–4) and one β subunit have been characterized to date, with a stoichiometry reported first as 3α/2β (Becker et al., 1988; Kuhse et al., 1993; Burzomato et al., 2003; Durisic et al., 2012) and later as 2α/3β (Grudzinska et al., 2005); although an stoichiometry of 4α/1β was recently reported (Yu et al., 2021; Zhu and Gouaux, 2021). The α2 subunit is expressed in the immature spinal cord, which switches to α1 in the adult, where the α1 and α3 subunits are expressed. In the adult brain the α1 and α3 subunits are mainly expressed; a4 has been demonstrated in mouse, chick and zebrafish, being a pseudogene in humans (Becker et al., 1988; Betz et al., 1993; Lynch, 2004). The adult retina expresses the four α subunits (Grünert, 2000; Haverkamp et al., 2004; Heinze et al., 2007; Sánchez-Chávez et al., 2017).

## Role of glycine during nervous system development

A variety of studies have focused to characterize the developmental expression of the glycine receptor and transporters as well as glycine immunoreactivity as means to recognize the process whereby cells adopt a glycinergic phenotype.

### Glycine levels

In cortical neuroepithelium, levels of glycine increase by twofold during embryogenesis, reach a peak around birth, and gradually decrease to about 60% during the first 2 weeks of postnatal development, time in which the GSC enzyme is highly expressed (Ichinohe et al., 2004).

In rodents, glycinergic neurons tend to appear during embryonic development in the rostral spinal cord, followed by increased expression caudally in the spinal cord and rostrally into the hindbrain, midbrain and retina (Van Den Pol and Gorcs, 1988; Allain et al., 2006). In the mice spinal cord, glycine immunoreactivity was found from E11.5 to E15.5 (Scain et al., 2010). Also, glycine immunoreactivity occurs in the inner retina since P1, and by P3-P5 in the outer retina; adult expression was found at P11 (Fletcher and Kalloniatis, 1997; Sharma et al., 2003).

Glycine immunoreactivity has also been examined in zebra fish (Moly et al., 2014), and chick embryos with positive staining, first observed at E8 in the dorsal and ventral spinal cord (Berki et al., 1995). In *Xenopus laevis* the first glycine positive cells appear in the rostral spinal cord and caudal hindbrain at stage 22, a few hours after the neural tube closes (Roberts et al., 1988).

### Glycine uptake

Glycine transporters appear early during embryonic brain development in rats. GlyT1 is predominant in the embryonic cortex and can be detected in radial glial cells (Jursky and Nelson, 1996).

Immunoreactivity for GlyT 1 and GlyT 2 glycine transporters was first observed at E10-12 in the midbrain floor plate. By E17, GlyT 1 expression is evident at the borders between the thalamus and hypothalamus, as well as at the border of the dorsal thalamus. GlyT 2 staining increases in the ventral spinal cord at E14 and in several brain regions at E17 (Jursky and Nelson, 1995; Lall et al., 2012). *In situ* hybridization studies in the zebra fish revealed the expression of GlyT1 and GlyT 2 in the rostral spinal cord at 18 and 20-h post-fertilization (Ganser and Dallman, 2009). Expression studies in *Xenopus laevis* showed the appearance of GlyT1 first in the proliferative ventricular layer of the hindbrain and the anterior spinal cord during early tail bud stages (stage 24) (Wester et al., 2008). In rat retina, pharmacological studies revealed the presence of both GlyT1 and GlyT 2 before final synaptogenesis has occurred (Salceda, 2006).

The role of GlyT 1 in glycine signaling was proved in E12.5 spinal cord cells, in which the decay rate of glycine current was increased by the presence of the GlyT 1 ALX-5407 inhibitor (Scain et al., 2010).

On the other hand, development of neurons in different regions of the brain is controlled by transcription factors. In this context, the expression of Ptf1a, Lbx1 and Pax2 transcription factors was described to be necessary for the expression of glycinergic phenotype in the spinal cord. Ptf1a, Lbx1, and Pax2 coordinate glycinergic and peptidergic transmitter phenotypes in dorsal spinal inhibitory neurons (Huang et al., 2008). Even more, transcription of GlyT 2 is activated by Pax2 (Batista and Lewis, 2008).

### Glycine receptors

During development, the GlyRs properties undergo molecular changes resulting in modifications of their physiological function; however, biochemical and molecular cloning studies have indicated heterogeneity of GlyRs subunits during development (Aguayo et al., 2004; Avila et al., 2013a).

Immunostaining for the α1 subunit is first seen in the rat spinal cord at E14, after which time the mRNA levels gradually increase in the ventral and dorsal horns until leveling off at P15. In the brain, α1 is detected at near adult levels by P5. The α2 subunit is expressed since E15 in the telencephalon, diencephalon, midbrain and cortex, and remains through early postnatal stages (P5) (Malosio et al., 1991; Watanabe and Akagi, 1995).

GlyRs α2 –homomers, are found throughout the CNS during development and its expression markedly decreases after birth (Watanabe and Akagi, 1995), switching for the expression of the adult, α1β heteromer (Lynch, 2004). The GlyRs subunit, α3, is observed until relatively late in development (P5), but it remains throughout life. The β subunit of GlyRs is first expressed at E14 in both the telencephalon and the ventral and dorsal horns of the spinal cord.

The postnatal rat retina shows GlyRs expression in the neuroblastic layer, while GlyR in the adults is only observed in the inner nuclear layer (INL) (Sassoè-Pognetto and Wässle, 1997). GlyR α2 subunit was found to be expressed in retinal progenitor cells at birth (Young and Cepko, 2004). Besides, a continuing increase of mRNA and protein expression of α1, α3, α4, and β subunits was found during postnatal retinal development, while α2 showed high levels in developing and adult retina (Sánchez-Chávez et al., 2017).

GlyRs are not only present during development but also functional. Whole-cell patch clamp motoneurons recordings in embryonic spinal cord show the first synaptic activity at E12.5; and demonstrate that radial cells release glycine, being the main source of it in the embryonic spinal cord (Scain et al., 2010). Likewise glycine elicited currents in different zones of the embryonic cortex were demonstrated at E19 (Flint et al., 1998). Moreover, it was shown that glycine application triggers a massive calcium influx in the upper-layer of pyramidal neurons at E17, effect that was blocked by strychnine and absent in the GlyR-knockout animals (Young-Pearse et al., 2006).

The expression of functional α2–containing GlyRs in cortical progenitors was demonstrated by whole-cell patch clamp recordings, where application of glycine triggered fast-activating currents. Moreover, *Glra2*-knockout mice show a reduced number of excitatory projection neurons in deep and upper layers of the cortex, leading to a modest reduction in brain size (Avila et al., 2013b,^2014^; Ávila et al., 2020).

### Glycine signaling

It is noteworthy that during development, glycine undergoes modifications of its kinetics and pharmacological properties (Aguayo et al., 2004). While glycine is an inhibitor neurotransmitter in the adult, it is excitatory in immature tissues (Rivera et al., 1999; Kandler et al., 2002; Kilb et al., 2002).

During development, chloride gradients change according to the expression of chloride transporters (Watanabe and Fukuda, 2015). In embryonic neurons, the sodium-potassium-chloride co-transporter (NKCC1) increases the intracellular concentration of chloride, then glycine binding to GlyRs causes a release of chloride ions and, therefore, induces a depolarization of the cell (Avila et al., 2014; Theisen et al., 2018). The switch from excitation to inhibition originates through the expression of the neural-specific potassium- chloride co-transporter 2 (KCC2), which actively reduces the intracellular concentration of chloride, transforming the opening of a chloride channel into a hyperpolarizing stimulus (Blaesse et al., 2006; Reynolds et al., 2008; Gonzalez-Islas et al., 2009; Liu and Wong-Riley, 2012). In other way, blocking GlyRs by strychnine decrease expression of KCC2 in ventral spinal networks without interfering with NKCC1; in addition, blockage of GlyRs led to decrease of KCC2 at the cell membrane (Allain et al., 2016), suggesting that glycine modulates KCC2.

In consequence, GlyRs activation during embryonic and early postnatal development induces a depolarization of the cell membrane (Flint et al., 1998; Kilb et al., 2002, 2008) which in turn may activate calcium influx (Platel et al., 2005). In fact, this depolarization activates voltage-sensitive sodium channels that subsequently activate sodium-sensitive calcium transporters, leading to the increase in intracellular calcium, which in turn may induce the release of glutamate (Kullmann et al., 2002; Platel et al., 2005; Brustein et al., 2013). In accordance with that, it was shown that applying glycine triggers a calcium influx in pyramidal and cortical neurons at E17 and E13, respectively (Platel et al., 2005). This effect was blocked by strychnine and disappears in the GlyR-knockout (KO) animals (Jimmy Zhou, 2001; Young-Pearse et al., 2006), supporting the specific effect of glycine through GlyRs.

Excitatory postsynaptic potentials produced by glycine have been observed since fetal to P7 in gerbils and rats. During this period neuronal growth occurs as well as the establishment of dendritic arbors (Sanes and Friauf, 2000). Glycinergic neurotransmission has been shown to influence neural maturation via modulating intracellular Ca^+2^ concentrations in the respiratory brainstem nuclei, hippocampus, and the lateral superior olive of the auditory system (Ben-Ari, 2001, 2013; Soria and Valdeolmillos, 2002; Ávila et al., 2020). In this regard, growing evidence is connecting the glycine induced depolarization with lateral superior olive (LSO) network maturation via modulating intracellular Ca^+2^ concentrations (Malenka and Nicoll, 1993; Sanes and Friauf, 2000; Kandler et al., 2002).

Similarly, the blockage of glycinergic transmission since the beginning of development by either embryonic glycine receptor knockdown (McDearmid et al., 2006), reversing the depolarizing chloride gradient by over expression of human KCC2 (Reynolds et al., 2008; Schwale et al., 2016), or by blocking GlyRs with strychnine (Côté and Drapeau, 2012) resulted in a selective reduction in the interneuron population with minimal changes in motoneurons and spinal sensory neuron populations.

This excitatory effect of glycine during embryonic development appears to be necessary for a broad range of neurogenic processes including formation and maturation of neuronal circuits (Ben-Ari, 2001; Ávila et al., 2020). Evidence indicate that GlyR α2 subunits are involved in the regulation of interneuron differentiation during spinal cord development (McDearmid et al., 2006) and synaptogenesis (Avila et al., 2013a,b; Lin et al., 2017; Ávila et al., 2020).

The role of glycinergic neurotransmission in the optimal balance of excitatory and inhibitory synaptic inputs during development is highlighted by the increase in dendritic arbors and dendritic spines found in motoneurons from gephyrin-deficient mice. These increases were associated with an increase of excitatory synaptic neurotransmission and a decrease of inhibitory neurotransmission (Banks et al., 2005; Fogarty et al., 2016). Also, it was demonstrated that GlyR α2 is needed for correct maturation and function of the glutamatergic striatum medium spiny neurons (Comhair et al., 2018).

In spite of that, glycine levels in the nervous tissue are assumed to be too low to allow normal neurotransmission during development (Van Den Pol and Gorcs, 1988; Zafra and Giménez, 2008) and it is hypothesized that the amino-sulfonic acid taurine, a partial agonist of GlyRs (Schmieden et al., 1992; Hussy et al., 1997; Mori et al., 2002; Jiang et al., 2004) may act as a ligand for the receptor. Supporting this hypothesis, it was shown that taurine function as a ligand for GlyR via non-synaptic signaling in the early neocortex (Flint et al., 1998). However, although the concentration of taurine progressively increases during embryogenesis and its levels are 10–20-fold higher than the levels of glycine and GABA (Benítez-Diaz et al., 2003), GlyRs in the developing cortex are 10 times less sensitive to taurine (Schmieden et al., 1992; Hussy et al., 1997; Okabe et al., 2004), strongly suggesting that glycine is acting on its own receptors.

On the other hand, glycine transporters may well play an important role controlling the extracellular level of glycine through GlyT1. In fact, it has been proposed that this could be the primarily role of GlyT1 during the spinal cord development, where this transporter is active in the removal of glycine from the extracellular compartment in extra synaptic locations (Gomeza et al., 2003).

In addition, glycine concentrations are influenced by the GCS that catalyzes the degradation of glycine and provides the developing brain with other metabolites, such as 5,10-methylenetetrahydrofolate, which is essential for DNA synthesis (Ichinohe et al., 2004).

## Role of glycine in cell proliferation and specification

Pioneering studies in the immature retina revealed a glycinergic transmission role in directing the proliferation of rod photoreceptor cells as well as in the light-dependent maturation of retinal ganglion and bipolar cells. Outstanding, overexpression of the α2 GlyR subunit leads to the development of a high percentage of rod photoreceptors at the expense of Muller glial cells (Young and Cepko, 2004).

Similarly, knockout of the α2 subunit at the onset of development reduces the number as well as the differentiation of spinal interneurons, thus affecting the formation of rhythm-generating networks (McDearmid et al., 2006). Moreover, α2-GlyRs were found to control the proliferation of progenitor cells during corticogenesis (Avila et al., 2014) and to promote the migration of cortical interneurons (Avila et al., 2013b).

Cells may be differentially affected depending on the type of GlyR subunit expressed as well as the cell type at different places compared to migrating interneurons (Ávila et al., 2020). In this context, glial cells can modulate neurotransmission by secretion of soluble factors; in fact, microglia secrete glycine and enhance NMDA receptor-mediated responses (Hayashi and Nakanishi, 2013).

Although glia are non-excitable, they express many of the same receptors for neurotransmitters, and these can induce membrane depolarization, increase in intracellular calcium, and proliferation (Domingues et al., 2010); indeed, GlyRs can modulate action potential conduction in white matter (Constantinou and Fern, 2009). Therefore, glial cells may also trough these receptors modulate synaptic development.

Remarkably, glycine has been showed to be related to the rapid cancer cell proliferation and could reverse the expression of aging phenotypes. This effect is thought to be related to glycine metabolism (Pan et al., 2021); glycine biosynthesis enzymes are more highly expressed in proliferating cells, where they are incorporated in purine nucleotides. In addition, depleting extracellular glycine or knocking down the SHMT2 glycine-synthesizing enzyme blocked rapid proliferation by prolonging the G1 phase of the cell cycle (Nguyen et al., 2002; Yang et al., 2018). Besides, α1 and α3 GlyR subunits were found to be expressed in human brain tumor biopsies (Förstera et al., 2014). Moreover, knockdown of α1 GlyR protein expression impaired the self-renewal capacity and tumorigenicity of GL261 glioma cells (Förstera et al., 2014), supporting a non- synaptic role of GlyRs. In this respect, outstandingly, α1 and α3 GlyR subunits were found to contain a nuclear localization signal in the large cytosolic loop domain (Melzer et al., 2010).

In addition, two inhibitors of the Wnt pathway (WIF1 and DKK1B) were upregulated upon GlyRs knockdown in neural stem cells (NSCs), suggesting that a Wnt-dependent neurogenic process could be silenced in NSCs when glycine signaling is impaired (Samarut et al., 2016, 2019). Moreover, the P53 tumor suppressor protein was upregulated upon GlyR knockdown, suggesting that cells might die in the absence of glycine signaling. Similarly, the activation of the Hedgehog signaling pathway reduces GlyT 2 expression *in vitro* in rodent primary spinal cord neurons or *in vivo* in zebrafish embryos (de la Rocha-Muñoz et al., 2021). These results define a link between development signaling pathways and glycine action.

### Pathophysiological consequences of glycinergic action during development

There is considerable evidence supporting the role of glycine on the CNS development. GlyRs are expressed in dorsal progenitors and migratory neurons, contributing to the cell cycle control, cell migration and morphological development (Tapia et al., 2000, 2001; Nimmervoll et al., 2011; Avila et al., 2013b; Avila et al., 2014), which impairs the formation of cortical circuits (Morelli et al., 2017). Therefore, the lack of GlyRs may affect the development process, including circuit formation that may lead to different disorders in adulthood (Ruediger and Bolz, 2007).

Indeed, defects in glycinergic signaling during neural development can result in the neurological motor disorders hyperekplexia, hypertonia, and episodic neonatal apnea (Lewis et al., 1998; Lape et al., 2012; Bode and Lynch, 2013). The hyperekplexia-causing mutations in *GLRA1* and *GLRB* result either in disrupted surface expression or altered glycine efficacy (Bode and Lynch, 2013).

Likewise, mutations in genes either encoding GlyRs (Piton et al., 2013; Pilorge et al., 2016), KCC2 (Merner et al., 2015), or the amino methyltransferase enzyme (AMT) involved in glycine degradation (Yu et al., 2013), were reported in patients affected by autism, supporting the glycine role in neurogenesis. Furthermore, recently Chen et al. (2022) using a combination of molecular modeling and electrophysiology recordings for four novel missense variants in GLRA2 associated with autism spectrum disorder (ASD), identified GLRA*2* as the cause of autism spectrum neurodevelopmental phenotypes. The missense variants cause either loss, gain or altered function of GlyR α2 subunit, enlightening the clinical forms associated with human ASD (Chen et al., 2022).

Similarly, failure in GCS activity leads to serious malformations, such as agenesis of the corpus callosum, gyral malformation, and cerebellar hypoplasia. Moreover, alternative splicing variants of GlyRs have been detected in patients suffering from temporal lobe epilepsy (Eichler et al., 2008). Also, GLRA2 knockout mice showed disruption of the excitation/inhibition balance, resulting in enhanced susceptibility to epileptic seizures (Morelli et al., 2017). Remarkably, pharmacological inhibition of GlyR α2 decreased the proliferation of hippocampal adult NSCs, and its genetic deletion leads to impaired and spatial memory in the adult mice (Lin et al., 2017).

Furthermore, the alpha-4 subunit and the β subunit were recently found to be expressed in mouse embryos, where were implicated in the regulation of embryo implantation (Nishizono et al., 2020).

## Conclusion

CNS development involved precise coordination of cell proliferation, differentiation and cell migration; in addition to an intrinsic genetic program, these processes are controlled by extracellular signals. Among these, neurotransmitters have been found to have an important role. In the adulthood, glycinergic neurotransmission is limited to the spinal cord, retina and few brain areas; however, functional GlyRs have been found almost everywhere in the developing brain.

Moreover a variety of evidence strongly support a relevant role of glycine signaling during development; even more, alterations in this signaling has been associated to pathologies in the adulthood (Eichler et al., 2008; Bode and Lynch, 2013; Piton et al., 2013; Pilorge et al., 2016; Morelli et al., 2017; Chen et al., 2022) supporting glycinergic function in proliferation and cell specification and circuit formation. Though, few studies have been carried out to analyze the mechanisms involved. Although these findings should be extended, they open new insights to understand the role of glycine during early neural development and its role in different pathologies, information which will improve adult brain healing.

## Author contributions

The author confirms being the sole contributor of this work and has approved it for publication.
